# Visually Lossless JPEG 2000 for Remote Image Browsing

**DOI:** 10.3390/info7030045

**Published:** 2016-07-15

**Authors:** Han Oh, Ali Bilgin, Michael Marcellin

**Affiliations:** 1 National Satellite Operation and Application Center, Korea Aerospace Research Institute (KARI); 169-84 Gwahak-ro, Yuseong-gu, Daejeon, 34133, Republic of Korea; 2 Department of Biomedical Engineering, The University of Arizona; 1127 E. James E. Rogers Way, Tucson, AZ, 85721, U.S.A.; 3 Department of Electrical and Computer Engineering, The University of Arizona; 1230 E. Speedway Blvd, Tucson, AZ, 85721, U.S.A.

**Keywords:** visually lossless coding, contrast sensitivity function, JPEG2000 Interactive Protocol (JPIP)

## Abstract

Image sizes have increased exponentially in recent years. The resulting high-resolution images are often viewed via remote image browsing. Zooming and panning are desirable features in this context, which result in disparate spatial regions of an image being displayed at a variety of (spatial) resolutions. When an image is displayed at a reduced resolution, the quantization step sizes needed for visually lossless quality generally increase. This paper investigates the quantization step sizes needed for visually lossless display as a function of resolution, and proposes a method that effectively incorporates the resulting (multiple) quantization step sizes into a single JPEG2000 codestream. This codestream is JPEG2000 Part 1 compliant and allows for visually lossless decoding at all resolutions natively supported by the wavelet transform as well as arbitrary intermediate resolutions, using only a fraction of the full-resolution codestream. When images are browsed remotely using the JPEG2000 Interactive Protocol (JPIP), the required bandwidth is significantly reduced, as demonstrated by extensive experimental results.

## 1. Introduction

With recent advances in computer networks and reduced prices of storage devices, image sizes have increased exponentially and user expectations for image quality have increased commensurately. Very large images, far exceeding the maximum dimensions of available display devices, are now commonplace. Accordingly, when pixel data are viewed at full resolution, only a small spatial region may be displayed at one time. To view data corresponding to a larger spatial region, the image data must be displayed at a reduced resolution, i.e., the image data must be downsampled. In modern image browsing systems, users can trade off spatial extent vs. resolution via zooming. Additionally, at a given resolution, different spatial regions can be selected via panning. In this regard, JPEG2000 has several advantages in the way it represents images. Owing to its wavelet transform, JPEG2000 supports inherent multi-resolution decoding from a single file. Due to its independent bit plane coding of “codeblocks” of wavelet coefficients, different spatial regions can be decoded independently, and at different quality levels [1]. Furthermore, using the JPEG2000 Interactive Protocol (JPIP), a user can interactively browse an image, retrieving only a small portion of the codestream [2–4]. This has the potential to significantly reduce bandwidth requirements for interactive browsing of images.

In recent years, significant attention has been given to visually lossless compression techniques, which yield much higher compression ratios compared with numerically lossless compression, while holding distortion levels below those which can be detected by the human eye. Much of this work has employed the contrast sensitivity function (CSF) in the determination of quantization step sizes. The CSF represents the varying sensitivity of the human eye as a function of spatial frequency and orientation, and is obtained experimentally by measuring the visibility threshold (VT) of a stimulus, which can be a sinusoidal grating [5,6] or a patch generated via various transforms, such as the Gabor Transform [7], Cortex Transforms [8–10], Discrete Cosine Transform (DCT) [11], or Discrete Wavelet Transform (DWT) [12,13].

The visibility of quantization distortion is generally reduced when images are displayed at reduced resolution. The relationship between quantization distortion and display resolution was studied through subjective tests conducted by Bae *et al.* [14]. Using the quantization distortion of JPEG and JPEG2000, they showed that users accept more compression artifacts when the display resolution is lower. This result suggests that quality assessments should take into account the display resolution at which the image is being displayed. Prior to this work, in [15], Li *et al.* proposed a vector quantizer which defines multiple distortion metrics for reduced resolutions and optimizes a codestream for multiple resolutions by switching the metric at particular bitrates. The resulting images show slight quality degradation at full resolution compared with images optimized only for full resolution, but exhibit considerable subjective quality improvement at reduced resolutions reconstructed at the same bitrate.

In [16], Hsiang and Woods proposed a compression scheme based on EZBC (Embedded image coding using ZeroBlocks of wavelet coefficients and Context modeling). Their system allows subband data to be selectively decoded to one of several visibility thresholds corresponding to different display resolutions. The visibility thresholds employed therein are derived from the model by Watson that uses the 9/7 DWT and assumes a uniform quantization distortion distribution [12].

This paper builds upon previous work to obtain a multi-resolution visually lossless coding method [17,18] that has the following distinct features:

1. The proposed algorithm is implemented within the framework of JPEG2000, which is an international image compression standard. Despite the powerful scalability features of JPEG2000, previous visually lossless algorithms using JPEG2000 are optimized for only one resolution [19–21]. This implies that if the image is rendered at reduced resolution, there are significant amounts of unnecessary information in the reduced resolution codestream. In this paper, a method is proposed for applying multiple visibility thresholds in each subband corresponding to various display resolutions. This method enables visually lossless results with much lower bitrates for reduced display resolutions. Codestreams obtained with this method are decodable by any JPEG2000 decoder.
2. Visibility thresholds measured using an accurate JPEG2000 quantization distortion model are used. This quantization distortion model was proposed in [22,23] and was developed for the statistical characteristics of wavelet coefficients and the dead-zone quantizer of JPEG2000. This model provides more accurate visibility thresholds than the commonly assumed uniform distortion model [16,19–21].
3. The proposed algorithm produces visually lossless images with minimum bitrates at the native resolutions inherently available in a JPEG2000 codestream as well as at arbitrary intermediate resolutions.
4. The effectiveness of the proposed algorithm is demonstrated for remotely browsing images using JPIP, described in Part 9 of the JPEG2000 standard [4]. Experimental results are presented for digital pathology images used for remote diagnosis [24] and for satellite images used for emergency relief. These high-resolution images with uncompressed file sizes of up to several Gigabytes (GB) each are viewed at a variety of resolutions from remote locations, demonstrating significant savings in transmitted data compared to typical JPEG2000/JPIP implementations.

This paper is organized as follows. Section II briefly reviews visually lossless encoding using visibility thresholds for JPEG2000 as used in this work. Section III describes the change in visibility thresholds of a subband when the display resolution is changed. A method is then presented to apply several visibility thresholds in each subband using JPEG2000. This method results in visually lossless rendering at minimal bitrates for each native JPEG2000 resolution. Section IV extends the proposed method to enable visually lossless encoding for arbitrary “intermediate” resolutions with minimum bitrates. In Section V, the performance of the proposed algorithm is evaluated. Finally, Section VI summarizes the work.

## 2. Visually Lossless JPEG2000 Encoder

Distortion in JPEG2000 results from differences between wavelet coefficient values at the encoder and the decoder that are generated by dead-zone quantization and mid-point reconstruction. This quantization distortion is then manifested as compression artifacts in the image, such as blurring or ringing artifacts, which are caused by applying the inverse wavelet transform. Compression artifacts have different magnitudes and patterns according to the subband in which the quantization distortion occurs. Thus, the visibility of quantization distortion varies from subband to subband. In [22,23], visibility thresholds (i.e., the maximum quantization step sizes at which quantization distortions remain invisible) were measured using an accurate model of the quantization distortion which occurs in JPEG2000. A visually lossless JPEG2000 encoder was proposed using these measured visibility thresholds. This section reviews this visually lossless JPEG2000 encoder which is the basis of the encoder proposed in subsequent sections of this paper.

### 2.1. Measurement of Visibility Thresholds

In this subsection, the measurement of the visibility thresholds employed in this work is summarized. Further details can be found in [22,23].

Assuming that the wavelet coefficients in the HL, LH, and HH subbands have a Laplacian distribution and that the LL subband has wavelet coefficients of a uniform distribution [25], the distribution of quantization distortion for the HL, LH, and HH subbands can be modeled by the probability density function (PDF)

**Figure 1:**
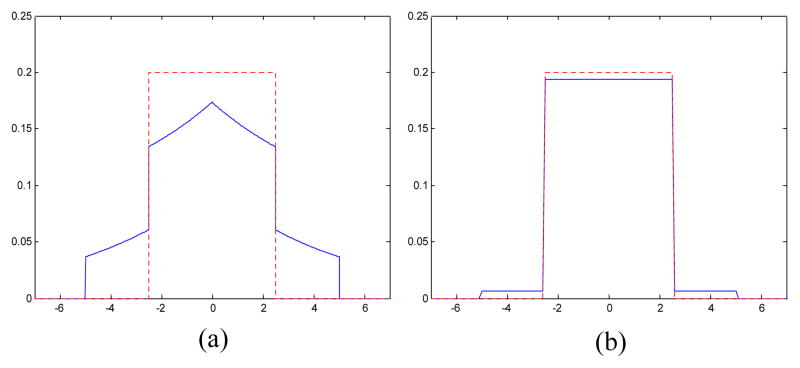
Probability density functions of: (a) the quantization distortion in HL, LH, and HH subbands (*σ*^2^ = 50, Δ = 5); and (b) the quantization distortion in the LL subband (*σ*^2^ = 2000, Δ = 5). The dashed lines represent the commonly assumed uniform distribution.

(1) f(d)={12σe-2∣d∣σ+1-p1Δ0≤∣d∣≤Δ212σe-2∣d∣σΔ2<∣d∣≤Δ0otherwise

where $p_{1}=\int_{-\Delta }^{\Delta }\frac{1}{\sqrt{2}\sigma }e^{-\frac{\sqrt{2}|y|}{\sigma }}dy=1-e^{-\sqrt{2}\Delta /\sigma }$. The parameters Δ and *σ* are the quantization step size and standard deviation of the wavelet coefficients, respectively. This model follows from the observation that wavelet coefficients in the dead-zone are quantized to 0 and coefficients outside the dead-zone yield quantization errors that are distributed approximately uniformly over (−Δ/2, Δ/2). The distribution of quantization distortion for the LL subband can be modeled by the PDF

(2) $$f(d)=\left\{ \begin{array}{cc} \frac{1}{\sqrt{12}\sigma }+\frac{1-p_{2}}{\Delta } & 0\leq \;|d|\;\leq \frac{\Delta }{2}\\ \frac{1}{\sqrt{12}\sigma } & \frac{\Delta }{2}<\;|d|\;\leq \Delta \\ 0 & \text{otherwise} \end{array}\right.$$

where $p_{2}=\Delta /\sqrt{3}\sigma$. These models are shown in Figure 1 for particular choices of quantization step size Δ and wavelet coefficient variance *σ*^2^.

The visibility thresholds for quantization distortion are obtained through psychophysical experiments with human subjects. A stimulus image is an RGB image with a gray background (*Y* = 128, *Cb* = 0, and *Cr* = 0 for 24-bit color images), obtained by applying the inverse wavelet transform and the inverse irreversible color transform (ICT)[1] to wavelet data containing quantization distortion. The quantization distortion is synthesized based on the JPEG2000 quantization distortion model given above for a given coefficient variance *σ*^2^. The stimulus image is displayed together with a uniformly gray image (which does not contain a stimulus), and a human subject is asked to select the stimulus image. The quantization step size used to generate the quantization distortion is adaptively varied by the QUEST staircase procedure in the Psychophysics Toolbox [26]. Through 32 iterations, the VT (the maximum quantization step size for which the stimulus remains invisible) is determined.

Unlike the conventional uniform quantization distortion model [12,13,16,19,20], indicated by the dashed line in Figure 1, the distribution of the quantization distortion is significantly affected by the variance of the wavelet coefficients. In general, an increase in coefficient variance leads to an increase in the visibility threshold. That is, larger distortions can go undetected when the coefficient variance is higher. For subband **b** = (*θ*, *k*), where *θ* ∈ {*LL*, *HL*, *LH*, *HH*} is the orientation of the subband and *k* is the DWT level, the visibility threshold can be modeled as a function of coefficient variance $\sigma_{b}^{2}$ by

(3) $$t_{b}(\sigma_{b}^{2})=u_{b}\cdot {\sigma_{b}}^{2}+v_{b}.$$

The parameters *u***_b_** and *v***_b_** were obtained from least-squares fits of thresholds measured via psycho-visual experiments for a variety of coefficient variances [23]. The resulting values for luminance are repeated here in Table 1. The chrominance thresholds were found to be insensitive to variance changes. That is, the corresponding values of *u***_b_** were found to be significantly smaller than those for the luminance thresholds. Thus, constant thresholds (independent of coefficient variance) were reported in [23] and repeated here in Table 2 for ease of reference. Similarly, a fixed threshold value of *t*_(_*_LL_*_,5)_ = 0.63 was reported for the LL subband of luminance components.

### 2.2. Visually Lossless JPEG2000 Encoder

This section summarizes the visually lossless JPEG2000 encoder of [22,23].

In JPEG2000, the *effective* quantization step size of each codeblock is determined by the initial subband quantization step size and the number of coding passes included in the final codestream. To ensure that the effective quantization step sizes are less than the VTs, the following procedure is followed for all luminance subbands except (LL,5). First, the variance $\sigma_{b,i}^{2}$ for the *i*-th codeblock ℬ*_i_* in subband **b** is calculated. Then $t_{b,i}(\sigma_{b,i}^{2})$ for that codeblock is determined using (3). During bit-plane coding, the maximum absolute coefficient error in the codeblock is calculated after each coding pass *z* as

(4) $$D^{\left(z\right)}=\max_{n\in B_{i}}\;\left(|y[n]-{\tilde{y}}^{\left(z\right)}[n]|\right)$$

where *ỹ*^(^*^z^*^)^[**n**] denotes the reconstructed value of *y*[**n**] using the quantization index *q̃*^(^*^z^*^)^[**n**], which has been encoded only up to coding pass *z*. Coding is terminated when *D*^(^*^z^*^)^ falls below the threshold $t_{b,i}(\sigma_{b,i}^{2})$. For the luminance (LL,5) and all chrominance subbands, the fixed VTs mentioned above are used as the initial subband quantization step size and all bit-planes are included in the codestream.

This JPEG2000 Part 1 complaint visually lossless encoder can significantly reduce computational complexity since bit-plane coding is not carried out for coding passes which do not contribute to the final codestream, and provides visually lossless quality at competitive bitrates compared to numerically lossless or other visually lossless coding methods in the literature. To further reduce the bitrate, masking effects which take into account locally changing backgrounds can be applied to the threshold values, at the expense of increased computational complexity [23].

In the following sections, the main contribution of the present work is described. In particular, visually lossless encoding as described in [22] and [23] is extended to the multi-resolution case. For simplicity, masking effects are not considered.

## 3. Multi-Resolution Visually Lossless JPEG2000

### 3.1. Multi-Resolution Visibility Thresholds

**Figure 2:**
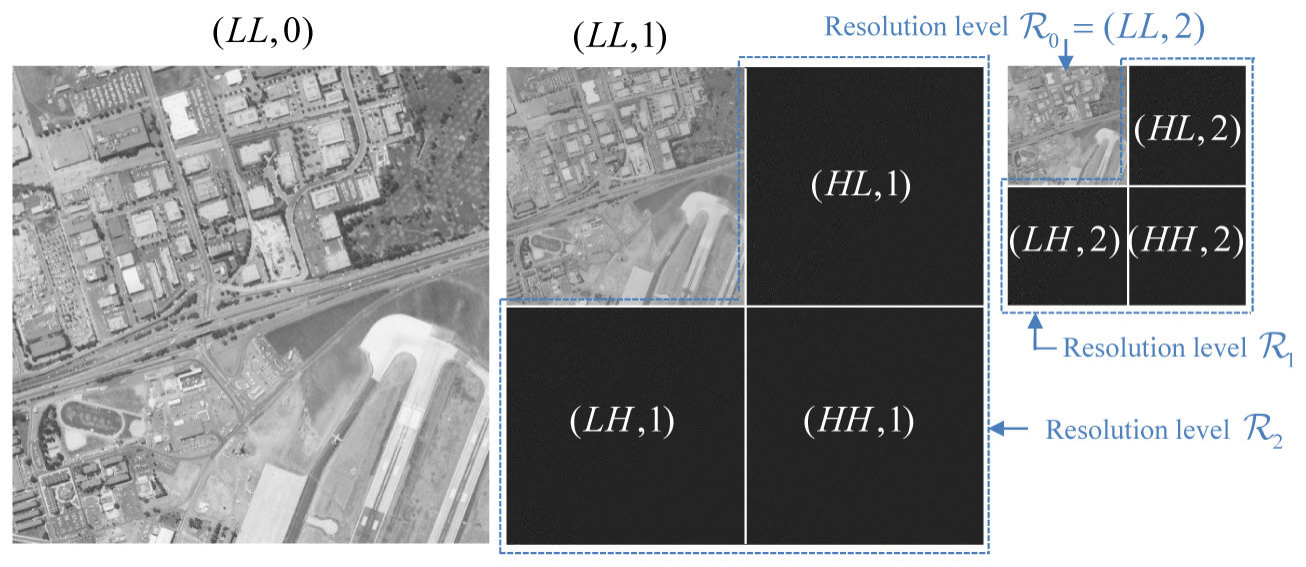
Resolution levels within a dyadic tree-structured subband decomposition with *K* = 2 levels.

JPEG2000, with *K* levels of dyadic tree-structured wavelet transform, inherently supports the synthesis of *K* + 1 different resolution images. As shown in Figure 2, the lowest resolution level ℛ_0_ corresponds to the lowest resolution image (*LL*, *K*). The next lowest resolution level ℛ_1_ = {(*HL*, *K*), (*LH*, *K*), (*HH*, *K*)} together with (*LL*, *K*) can be used to render the next to lowest resolution image (*LL*, *K* − 1). Continuing in this fashion, resolution level ℛ*_r_* together with the image (*LL*, *K* − (*r* − 1)) can be used to synthesize the image (*LL*, *K* − *r*), for 1 ≤ *r* ≤ *K*, with the full resolution image denoted by (*LL*, 0).

In what follows, it is assumed that images are always displayed so that each “image pixel” corresponds to one “monitor pixel.” Under this assumption, when displayed as an image, (*LL*, *K* − *r*) can be thought of as masquerading as a full resolution image. The subbands of resolution level ℛ*_r_* can then be seen to play the role of the highest frequency subbands, normally played by ℛ*_K_*. For example, in Figure 2, it can be seen that ℛ_1_ = {(*HL*, 2), (*LH*, 2), (*HH*, 2)} contains the highest frequency subbands of the “image” (*LL*, 1). Similarly, the subbands of resolution level ℛ*_r_*_−1_ play the role normally played by those from resolution level ℛ*_K_*_−1_, and so on. In general, when displaying image (*LL*, *K* − *r*), resolution level ℛ*_j_* behaves as resolution level ℛ*_j_*_+(_*_K_*_−_*_r_*_)_, 0 < *j* ≤ *r*. The lowest resolution level ℛ_0_ = (*LL*, *K*) behaves as (*LL*, *r*). Therefore, to have visually lossless quality of the displayed image (*LL*, *K* − *r*), the visibility thresholds used for ℛ*_j_* should be those normally used for ℛ*_j_*_+(_*_K_*_−_*_r_*_)_. It then follows that when reduced resolution image (*LL*, *K* − *r*) is displayed, the visibility threshold for subband **b** = {*θ*, *k*} is given by

(5) $${\tilde{t}}_{b,r}(\sigma_{b}^{2})=t_{\tilde{b}}(\sigma_{b}^{2})\;\text{for}\;(K-r)<k\leq K$$

where **b̃** = (*θ*, *k* − (*K* − *r*)) and $t_{b}(\sigma_{b}^{2})$ is the threshold normally used for subband **b** to achieve visually lossless quality when the full resolution image (*LL*, 0) is displayed. As usual, subbands with *k* ≤ (*K* − *r*) are discarded when forming (*LL*, *K* − *r*). This can be considered as setting their thresholds to infinity.

For *K* = 5, (3) and (5) together with Tables 1 and 2 can then be used to determine appropriate visibility thresholds for all subbands except (*LL*, 5). As mentioned in the previous paragraph, and indicated by (5), (*LL*, 5) plays the role of (*LL*, *r*) when the image (*LL*, 5 − *r*) is displayed. The work of [22] and [23] provided threshold values only for (*LL*, 5) and not for (*LL*, *k*), *k* < 5. Therefore, in the work described herein, additional thresholds are provided for (*LL*, *k*), 0 ≤ *k* ≤ 5. For each such subband, thresholds were measured for a wide range of $\sigma_{b}^{2}$. Least squares fitting was then performed to obtain the parameters *u*_(_*_LL_*_,_*_k_*_)_ and *v*_(_*_LL_*_,_*_k_*_)_ for the model

(6) $$t_{\left(LL,k\right)}(\sigma_{\left(LL,k\right)}^{2})=u_{\left(LL,k\right)}\log_{10}\sigma_{\left(LL,k\right)}^{2}+v_{\left(LL,k\right)}.$$

The resulting values for *u*_(_*_LL_*_,_*_k_*_)_ and *v*_(_*_LL_*_,_*_k_*_)_ are listed in Table 3. Substituting (6) into (5) yields the threshold value to be used for (*LL*, *K*) when displaying image (*LL*, *K* − *r*). Specifically,

(7) $${\tilde{t}}_{(LL,K),r}(\sigma_{\left(LL,K\right)}^{2})=u_{\left(LL,r\right)}\log_{10}\sigma_{\left(LL,K\right)}^{2}+v_{\left(LL,r\right)}.$$

Recall that a fixed threshold was employed for (*LL*, 5) of the luminance component in [22] and [23]. In contrast, thresholds depending on codeblock variances are employed here for (*LL*, *k*), 0 ≤ *k* ≤ 5. This is due to the large number of codeblocks in these subbands exhibiting extreme variability in coefficient variances. Fixed thresholds still suffice for the chrominance components. Table 4 shows the chrominance threshold values *t*_(_*_LL_*_,_*_k_*_)_ measured at an assumed typical variance of *σ*^2^ = 150.

**Figure 3:**
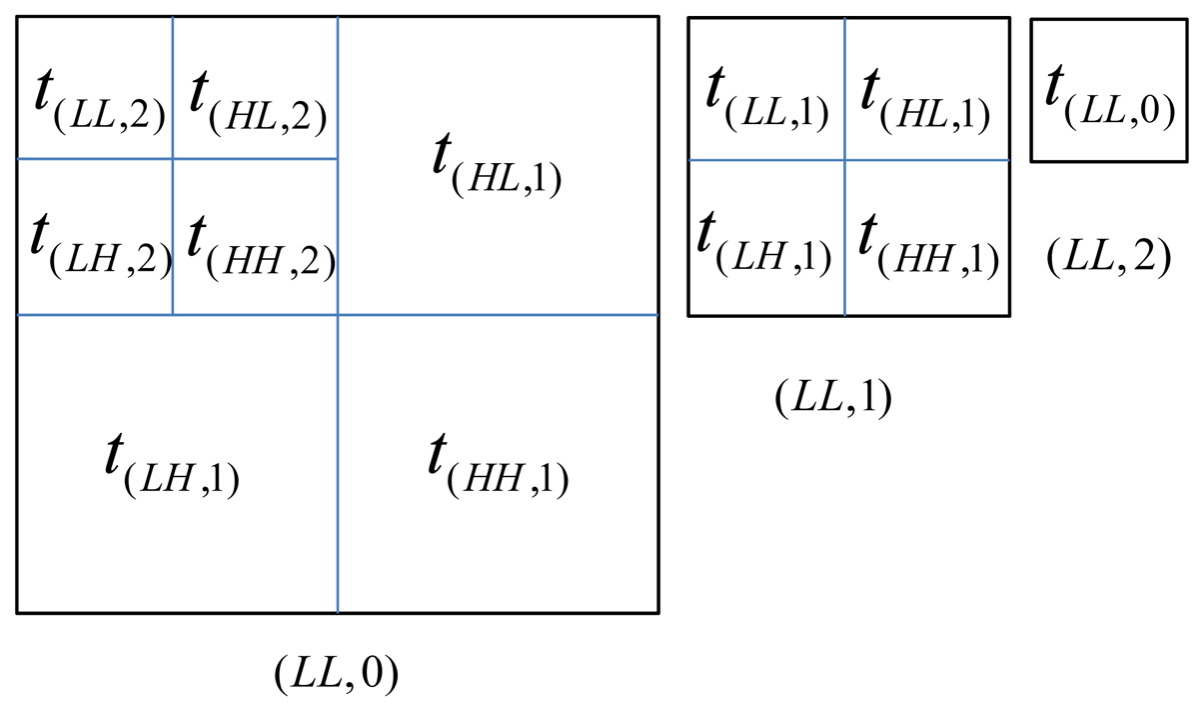
Visibility thresholds at three display resolutions (*K* = 2).

Figure 3 illustrates the discussion above for *K* = 2. When the full resolution image (*LL*, 0) (*r* = 2) is displayed, subband (*θ*, *k*) requires threshold *t*_(_*_θ_*_,_*_k_*_)_ for visually lossless quality. However, when the one-level reduced resolution image (*LL*, 1) (*r* = 1) is displayed, the four subbands with *k* = 2 which previously needed thresholds *t*_(_*_θ_*_,2)_ now require thresholds *t*_(_*_θ_*_,1)_. Similarly, when the lowest resolution image (*LL*, 2) (*r* = 0) is displayed, threshold *t*_(_*_LL_*_,0)_ is applied.

### 3.2. Visually Lossless Quality Layers

From Tables 1 through 4, it can be seen that visibility threshold values increase monotonically as the resolution level increases. This implies that the threshold for a given subband increases as the display resolution is decreased. Now, consider the case when an image is encoded with visibility thresholds designed for the full resolution image. Consider further forming a reduced resolution image in the usual manner by simply dropping the unneeded high frequency subbands. The (lower frequency) subbands still employed in image formation can be seen as being encoded using smaller thresholds than necessary, resulting in inefficiencies. Larger thresholds could be employed for these subbands resulting in smaller codestreams. In what follows, we describe how the (quality) layer functionality of JPEG2000 can be used to apply multiple thresholds, each optimized for a different resolution.

JPEG2000 layers are typically used to enable progressive transmission, which can increase the perceived responsiveness of remote image browsing. That is, when progressive transmission is used, the user often perceives that useful data are rendered faster for the same amount of data received. In JPEG2000, each codeblock of each subband of each component contributes 0 or more consecutive coding passes to a layer. Beginning with the lowest (quality) layer 𝒬_0_, image quality is progressively improved by the incremental contributions of subsequent layers. In typical JPEG2000 encoder implementations, each layer is constructed to have minimum mean squared error (MSE) for a given bitrate, with the aid of post-compression rate-distortion optimization (PCRD-opt) [27]. More layers allow finer grained progressivity and thus more frequent rendering updates of displayed imagery, at the expense of a modest increase in codestream overhead. To promote spatial random access, wavelet data from each resolution level are partitioned into spatial regions known as precincts. Precinct sizes are arbitrary (user selectable) powers of 2 and can be made so large that no partitioning occurs, if desired. All coding passes from one layer that belong to codeblocks within one precinct of one resolution level (of one image component) are collected together in one JPEG2000 packet. These packets are the fundamental units of a JPEG2000 codestream.

In the work described here, layers are tied to resolutions so that layer 𝒬_0_ provides “just” visually lossless reconstruction of (*LL*, *K*). The addition of layer 𝒬_1_ enables just visually lossless reconstruction of (*LL*, *K* − 1), and so on. More precisely, layer 𝒬*_l_* is constructed so that when layers 𝒬_0_ through 𝒬*_l_* are decoded, the maximum absolute quantization error, *D*^(^*^z^*^)^ is just smaller than the visibility threshold ${\tilde{t}}_{b,l}(\sigma_{b}^{2})$ for every codeblock in every resolution level ℛ*_l_*, 0 ≤ *l* ≤ *r*. In this way, when image (*LL*, *K* − *r*) is decoded using only layers 𝒬*_l_*, 0 ≤ *l* ≤ *r*, all relevant codeblocks are decoded at the quality corresponding to their appropriate visibility thresholds.

**Figure 4:**
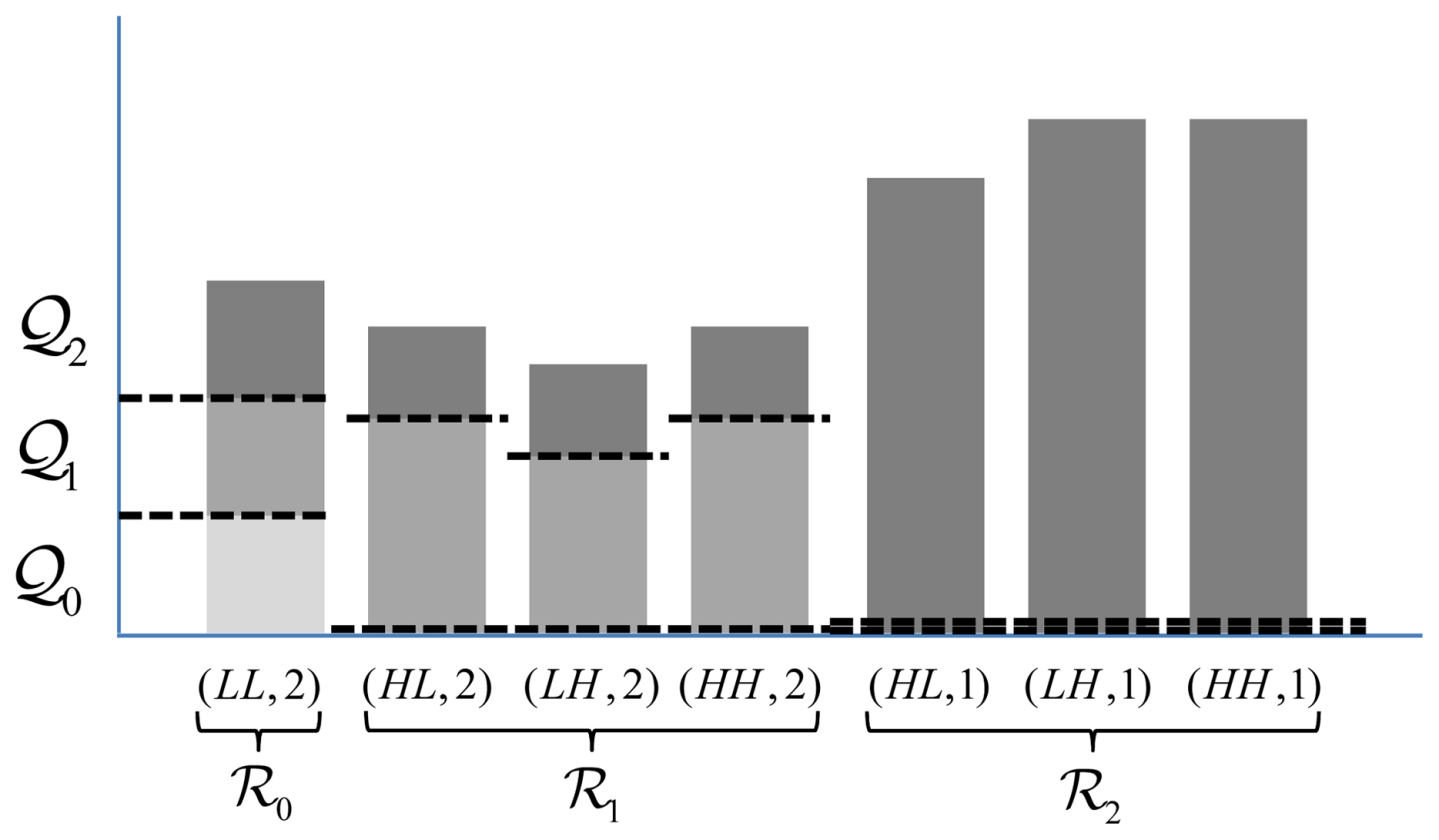
Quality layers for three display resolutions (*K* = 2).

Figure 4 shows an example of quality layers generated for three display resolutions (*K* = 2). The lowest resolution image (*LL*, 2) needs only layer 𝒬_0_ for visually lossless reconstruction. At the next resolution, an additional layer 𝒬_1_ is decoded. That is, image (*LL*, 1) is reconstructed using both 𝒬_0_ and 𝒬_1_. At full resolution, the information from the final layer 𝒬_2_ is incorporated. It is worth reiterating that the JPEG2000 codestream syntax requires that every codeblock contribute 0 or more coding passes to each layer. The fact that in the proposed scheme, each codeblock in ℛ_1_ and ℛ_2_ contribute 0 coding passes to 𝒬_0_ (and that ℛ_2_ contributes 0 coding passes to 𝒬_1_) is indicated in the figure. This results in a number of empty JPEG2000 packets. The associated overhead is negligible, since each empty packet occupies only one byte in the codestream.

The advantages of the proposed scheme are clear from Figure 4. Specifically, when displaying (*LL*, 2), a straightforward treatment would discard (*HL*, 2) through (*HH*, 1) for considerable savings. However, it would retain unneeded portions (𝒬_1_ and 𝒬_2_) of (*LL*, 2). Similarly, when displaying (*LL*, 1), a straightforward treatment would discard (*HL*, 1) through (*HH*, 1). However, it would still include unneeded data in the form of 𝒬_2_ for (*LL*, 2) through (*HH*, 2). By discarding these unneeded data, the proposed scheme can achieve significant savings.

## 4. Visibility Thresholds For Downsampled Images

In the previous section, visibility thresholds and visually lossless encoding were discussed for the native resolutions inherently available in a JPEG2000 codestream (all related by powers of 2). In this section, “intermediate” resolutions are considered. Such resolutions may be obtained via resampling of adjacent native resolution images. To obtain an image with a resolution between (*LL*, *K* − *r* + 1) and (*LL*, *K* − *r*), there are two possibilities: 1) upscaling from the lower resolution image (*LL*, *K* − *r* + 1); or 2) downscaling from the higher resolution image (*LL*, *K* − *r*). It is readily apparent that upscaling a decompressed version of (*LL*, *K* − *r* + 1) will be no better than an upscaled (interpolated) version of an uncompressed version of (*LL*, *K* − *r* + 1). Visual inspection confirms that this approach does not achieve high quality rendering. Thus, in what follows, we consider only downsampling of the higher resolution image (*LL*, *K* − *r*). A decompressed version of (*LL*, *K* − *r*) may be efficiently obtained by decoding 𝒬*_l_*, 0 ≤ *l* ≤ *r*, as described in the previous section. However, even this method decodes more data than required for the rendering of imagery downsampled from (*LL*, *K* − *r*). In what follows, the determination of the visibility thresholds for downscaled images is discussed.

Measurement of visibility thresholds for downscaled images is conducted in the fashion as described previously, but stimulus images are resampled by a rational factor of *I*/*D* before display. Resampling is performed in the following order: insertion of *I* zeros between each pair of consecutive samples, low-pass filtering, and decimation by *D*. In this experiment, visibility thresholds are measured for three intermediate resolutions below each native resolution. The resampling factors employed are 0.60 = 0.5(1.2), 0.72 = 0.5(1.2)^2^, and 0.864 = 0.5(1.2)^3^. These factors are applied as downscaling factors from the one-level higher resolution image (*LL*, *K* − *r*) for 0 ≤ *r* ≤ *K*. On the other hand, they can be thought of (conceptually) as resulting in successive 20% increases in resolution from (*LL*, *K* − *r* + 1). We begin by considering the subsampling of the full resolution image (*LL*, 0).

Table 5 lists measured visibility thresholds *t̄***_b_**_,_*_n_* for *K* = 5. The subscript *n* has been added to the notation to indicate the subsampling factor. Values, of *n* ∈ {1, 2, 3} correspond to subsampling factors of 0.5(1.2)*^n^*, while *n* = 4 corresponds to a subsampling factor of 1.0 (no subsampling). As explained in Section II-A, quantization distortion varies with the variance of wavelet coefficients. However, the values in Table 5 were only measured for a fixed typical variance per subband. Psychovisual testing for all possible combinations of subbands, resolutions, and variances is prohibitive. Instead, the thresholds in Table 5 are adjusted to account for changes in variance as follows: In the case of chrominance components, the thresholds are not adjusted because, as before, chrominance threshold values are insensitive to coefficient variance (i.e., $t_{b,n}(\sigma_{b}^{2})≅{\bar{t}}_{b,n}$). On the other hand, the luminance subbands are significantly affected by variance differences. For these subbands, the visibility threshold *t***_b_**_,4_(*σ*^2^) corresponds to the non-subsampled case, and is given in (3) as before. Thresholds for *n* ∈ {1, 2, 3} are then obtained via

(8) $$t_{b,n}(\sigma^{2})={\bar{t}}_{b,n}\;\left\{\frac{t_{b,4}(\sigma^{2})}{{\bar{t}}_{b,4}}\right\}.$$

From (8) and Table 5, $t_{b,n}(\sigma_{b}^{2})>t_{b,n+1}(\sigma_{b}^{2})$ for *n* ∈ {1, 2, 3} resulting in coarser quantization and smaller files.

Extension to dowsampling starting from any native (power of 2) resolution results from updating (5) to yield

(9) $${\tilde{t}}_{b,n,r}(\sigma_{b}^{2})=t_{\tilde{b},n}(\sigma_{b}^{2})\;\text{for}\;(K-r)<k\leq K.$$

Threshold ${\tilde{t}}_{b,n,r}(\sigma_{b}^{2})$ is then the maximum quantization step size for subband **b** that provides visually lossless quality when image (*LL*, *K* − *r*) is downscaled by downsampling factor *n*, resulting in minimum file size for that downsampling. Table 6 contains the necessary values for *t̄***_b_**_,_*_n_* for **b̄** = (*LL*, *k*), 0 ≤ *k* < 5.

The thresholds defined above are applied in JPEG2000 by defining 4(*K* + 1) layers – one for each resolution to be rendered (each native resolution together with its three subsampled versions, *n* = 1, 2, 3). Layer 𝒬*_l_* is then constructed such that codeblock *i* from subband **b** contains only all coding passes up to the first coding pass *z* that ensures the maximum quantization error *D*^(^*^z^*^)^ falls just below the threshold ${\tilde{t}}_{b,n(l),r(l)}(\sigma_{b,i}^{2})$, where *r*(*l*) = ⌊*l*/4⌋ and *n*(*l*) = *l* − 4*r*(*l*) + 1 and $\sigma_{b,i}^{2}$ is the variance of codeblock *i* from subband **b**. Decoding from 𝒬_0_ to 𝒬*_l_* then ensures visually lossless reconstruction of image (*LL*, *K* − *r*(*l*)) when downscaled by downsampling factor *n*(*l*).

A visually lossless image with completely arbitrary resolution scale *p* ∈ (0, 1] with respect to the full resolution image (*LL*, 0) can be obtained by calculating *r* = *K* − ⌊ − log_2_ *p* ⌋, *n* = ⌈(log_2_ *p* + ⌊−log_2_ *p*⌋ + 1)/log_2_ 1.2⌉, and *l* = *n* + 4*r* − 1. The decoded version of image (*LL*, *K* − *r*), using layers 𝒬_0_ through 𝒬*_l_*, then provides enough quality so that appropriate resampling yields a visually lossless image with resolution scale *p*.

## 5. Experimental Results

### 5.1. Multi-Resolution Visually Lossless Coding

The proposed multi-resolution visually lossless coding scheme was implemented in Kakadu v6.4 [28]. Experimental results are presented for seven digital pathology images and eight satellite images. All of the images are 24-bit color high resolution images ranging in size from 527 MB (13165 × 14000) to 3.23 GB (39912 × 29032). Each image is identified with *pathology* or *satellite* together with an index, e.g., *pathology 1* or *satellite 3*. Recent technological developments in digital pathology allow rapid processing of pathology slides using array microscopes [24]. The resulting high-resolution images (referred to as virtual slides) can then be reviewed by a pathologist either locally or remotely over a telecommunications network. Due to the high resolution of the imaging process, these images can easily occupy several GBytes. Thus, remote examination by the pathologist requires efficient methods for transmission and display of images at different resolutions and spatial extents at the reviewing workstation. The satellite images employed here show various locations on Earth before and after natural disasters. The images were captured by the GeoEye-1 satellite, at 0.5 meter resolution from 680 km in space, and were provided for the use of relief organizations. These images are also so large that fast rendering and significant bandwidth savings are essential for efficient remote image browsing.

In this work, “reference images” corresponding to reduced native resolution images (*LL*, 5 −*r*), *r* = 0, 1, ..., 4 were created using the 9/7 DWT without quantization or coding. Reference images for intermediate resolutions were obtained by downscaling the next (higher) native resolution reference image. In what follows, the statement that a decompressed reduced resolution image is visually lossless means that it is visually indistinguishable from its corresponding reference image.

To evaluate the compression performance of the proposed method, each image was encoded using three different methods. The first method is referred to here as the 6-layer method. As the name suggests, codestreams from this method employ six layers to apply the appropriate visually lossless thresholds for each of six native resolution images (*LL*, 5 − *r*), *r* = 0, 1, ..., 5 (*K* = 5). The second method, referred to as the 24-layer method, uses a total of 24 layers to provide visually lossless quality at each of the six native resolutions, plus three intermediate resolutions below each native resolution. The third method, used as a benchmark, employs the method from [22] to yield a visually lossless image optimized for display only at full resolution. The codestream for this method contains a single layer, so this benchmark is referred to as the single-layer method. To facilitate spatial random access, all images were encoded using the CPRL progression order with precincts of size 128 *×* 128 at each resolution level.

**Figure 5:**
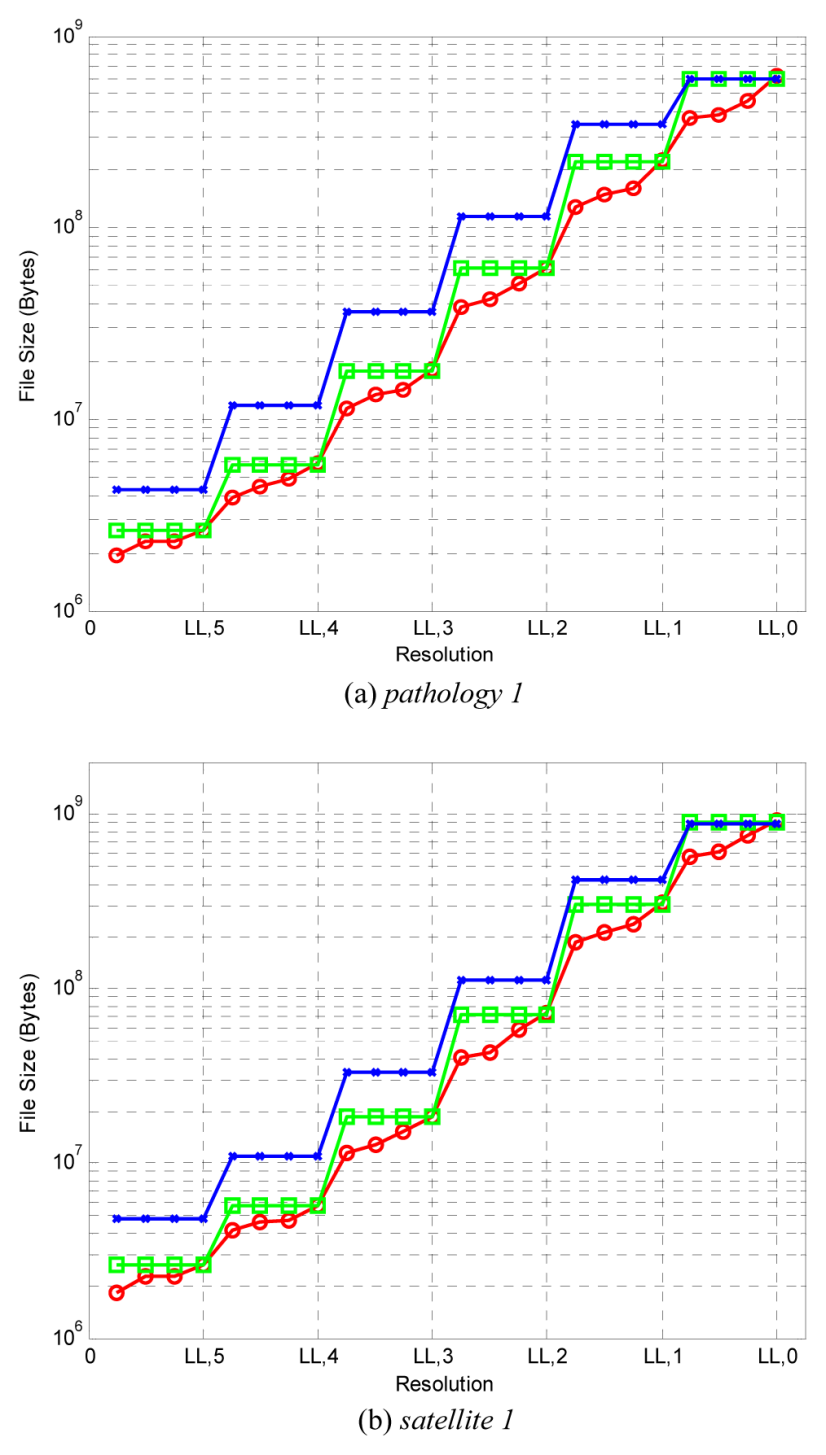
Number of bytes required for decompression by the three described coding methods. The single-layer, 6-layer, and 24-layer methods at each resolution are denoted by crosses, rectangles, and circles, respectively.

Figure 5 compares the number of bytes that must be decoded (transmitted) for each of the three coding methods to have visually lossless quality at various resolutions. Results are presented for one image of each type. Graphs for other images are similar. The number of bytes for the single-layer, 6-layer, and 24-layer methods at each resolution are denoted by crosses, rectangles, and circles, respectively. As expected, the curves for the single-layer method generally lie above those of the 6-layer method, which in turn generally lie above those of the 24-layer method. It is worth noting that the vertical axis employs a logarithmic scale, and that gains in compression ratio are significant for most resolutions.

Table 7 lists bitrates obtained (in bits-per-pixel with respect to the dimensions of the full-resolution images) averaged over all 15 test images. From this table, it can be seen that the 6-layer method results in 39.3%, 50.0%, 48.1%, 42.1%, and 31.0% smaller bitrate compared to the single-layer method for reduced resolution images (*LL*, 5 − *r*), *r* = 0, 1, 2, 3, and 4, respectively. In turn, for the downsampled images (*LL*, 5 − *r*), *n* = 1, *r* = 0, 1, 2, 3, 4, and 5, the 24-layer method provides 25.0%, 30.3%, 35.5%, 39.1%, 39.1%, and 36.7% savings in bitrate, respectively compared to the 6-layer method. These significant gains are achieved by discarding unneeded codestream data in the relevant subbands in a precise fashion, while maintaining visually lossless quality in all cases. Specifically, the 6-layer case can discard data in increments of one layer out of 6, while the 24-layer method can discard data in increments of one layer out of 24. In contrast, the single-layer method must read *all* data in the relevant subbands.

**Figure 6:**
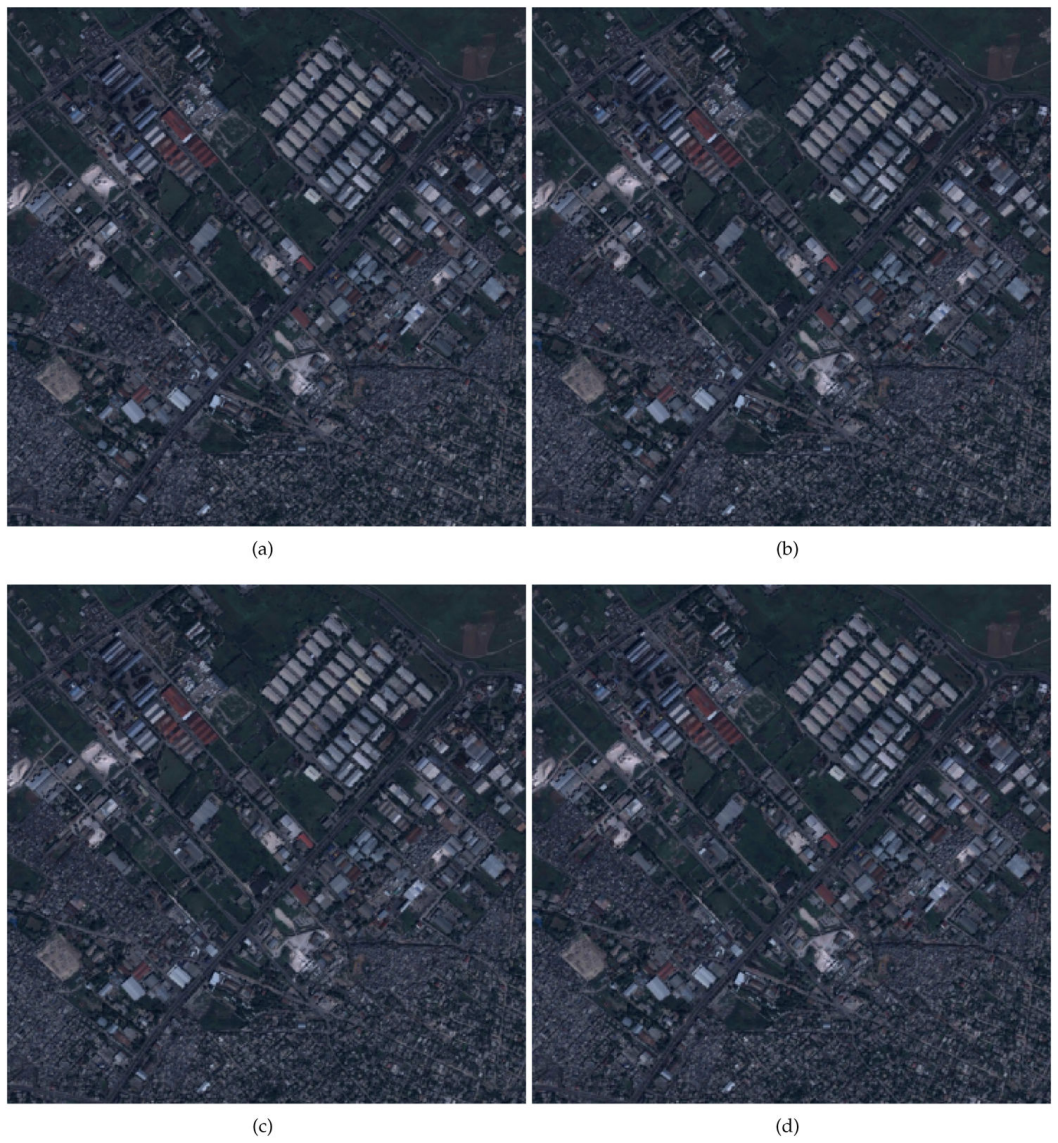
*Satellite 2* image rendered at resolution *p* = 0.09. (a) reference, (b) single-layer method, (c) 6-layer method, and (d) 24-layer method. The images are cropped to 312 *×* 312 after rendering to avoid rescaling during display. The images should be viewed with PDF viewer scaling set to 100%. Satellite image courtesy of GeoEye.

Figure 6 shows crops of the *satellite 2* image reconstructed at resolution *p* = 0.09 for the three coding methods. Each of these images is downscaled from a version of (*LL*, 3) (*p* = 0.125). Specifically, for the single-layer method, the image is downscaled from all (*LL*, 3) data which amounts to 5.30 bpp, relative to the reduced resolution dimensions. For the proposed methods, the image is downscaled from 3 of 6 layers and 10 of 24 layers of (*LL*, 3) data which amounts to 2.98 bpp and 2.09 bpp, respectively. Although the the proposed methods offer significantly lower bitrates, all three resulting images have the same visual quality. That is, they are all indistinguishable from the reference image.

Although this multi-layer method provides significant gains for most resolutions, there exist a few (negligible) losses for some resolutions. Specifically, the 6-layer case is slightly worse than the single-layer case for (*LL*, 0) as well as for the three intermediate resolutions immediately below (*LL*, 0). The average penalty in this case is 0.72%. Similarly, the 24-layer case is slightly worse than the 6-layer case at each of the six native resolutions. The average penalty for (*LL*, 5 − *r*), *r* = 0, 1, 2, ..., 5 is 0.19%, 0.48%, 0.88%, 1.30%, 1.89%, and 2.23%, respectively. These minor drops in performance are due to the codestream syntax overhead associated with including more layers.

As mentioned previously, the layer functionality of JPEG2000 enables quality scalability. As detailed in the previous paragraph, for certain isolated resolutions, the single-layer method provides slightly higher compression efficiency as compared to the 6-layer and 24-layer methods. However, it provides *no quality scalability and therefore no progressive transmission capability*. To circumvent this limitation, layers could be added to the so called single-layer method. To this end, codestreams from the single-layer method were partitioned into six layers. The first five layers were constructed via the arbitrary selection of five rate-distortion slope thresholds, as normally allowed by the Kakadu implementation. The six layers together yield exactly the same decompressed image as the single-layer method. In this way, the “progressivity” is roughly the same as the 6-layer method, but visually lossless decoding of reduced resolution images is not guaranteed for anything short of decoding all layers for the relevant subbands. Table 8 compares the bitrates for decoding (*LL*, 0) under the proposed 6-layer visually lossless coding method vs. the one-layer method with added layers for the *pathology 3* and *satellite 6* images. As seen from the table, the results are nearly identical. Results for other images as well as for 24-layers are similar. Thus, the overhead associated with the proposed method is no more than that needed to facilitate quality scalability/progressivity.

### 5.2. Validation Experiments

To verify that the images encoded with the proposed scheme are visually lossless at each resolution, a three-alternative forced-choice (3AFC) method was used, as in [23]. Two reference images and one compressed image were displayed side by side for an unlimited amount of time, and the subject was asked to choose the image that looked different. The position of the compressed image was chosen randomly. In the validation experiments, all compressed images were reconstructed from codestreams encoded with the 24-layer method. Validating the 24-layer method suffices since this method uses the same or larger threshold values than those of the 6-layer or single-layer method at each resolution. In other words, the 24-layer method never uses less aggressive quantization than the other two methods. All 15 images were used in the validation study. For each image, compressed images and reference images were generated at two native resolutions, (*LL*, 5) (*p* = 0.03125) and (*LL*, 2) (*p* = 0.25), as well as two intermediate resolutions corresponding to (*LL*, 3), *n* = 2 (*p* = 0.09) and (*LL*, 0), *n* = 2 (*p* = 0.72). Full resolution images (*p* = 1.0) were validated in [23]. These five resolutions form a representative set spread over the range of possible values. After decompression, all images were cropped to 512 *×* 512 at a random image location so that three copies fit side by side on a Dell U2410 LCD monitor with display size 1920 *×* 1200. The validation experiment consisted of four sessions – one for each resolution tested. In each session, all 15 images were viewed five times at the same resolution (75 trials for each subject) in random order. Subjects were allowed to rest between sessions. Twenty subjects, who are familiar with image compression and have normal or corrected-to-normal vision, participated in the experiment. Each subject gave a total of 300 responses over the four sessions. The validation was performed under the same viewing conditions as in [23] (Dell U2410 LCD monitor, ambient light, a viewing distance of 60 cm). During the experiment, no feedback was provided on the correctness of choices.

If the compressed image is indistinguishable from the reference image, the correct response should be obtained with a frequency of 1/3. Table 9 shows the statistics obtained and t-test results with a test value of 1/3. It can be seen that the hypothesis that the responses were randomly chosen could not be rejected at the 5% significance level for each of the four sessions. Based on these results, it is claimed that the proposed coding method provides visually lossless quality at every tested resolution.

It is worth noting that for medical images, such as the pathology images employed as part of the test set herein, visually lossless compression may not be the most relevant goal. Indeed, “diagnostically lossless” compression may be more interesting for this type of imagery. Indeed, our ongoing work is focused on the maximization of compression efficiency while maintaining diagnostic accuracy.

### 5.3. Performance Evaluation Using JPIP

JPIP is a connection-oriented network communication protocol that facilitates efficient transmission of images using the characteristics of scalable JPEG2000 codestreams. A user can interactively browse spatial regions of interest, at desired resolutions, by retrieving only the corresponding minimum required portion of the codestream.

**Figure 7:**
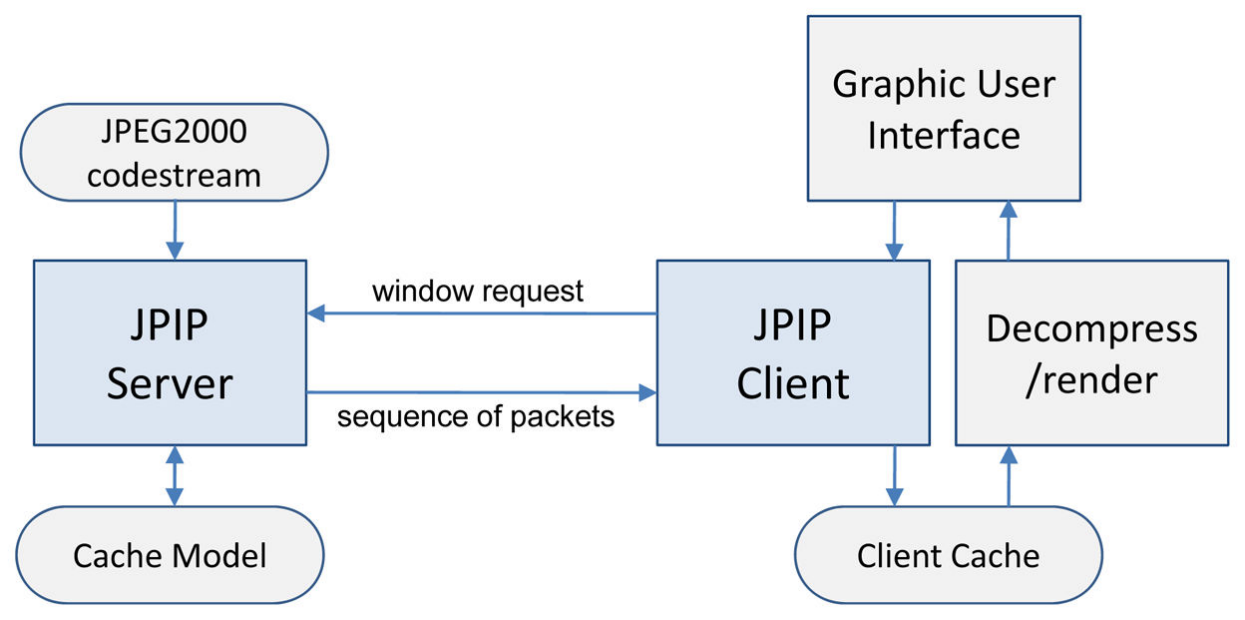
Client-server interaction in a JPIP remote image browsing system.

Figure 7 shows a block diagram of a JPIP remote image browsing system. First, the client requests the spatial region, resolution, number of layers, and image components of interest using a simple descriptive syntax to the server. In response to the client request, the server accesses the relevant packets from the JPEG2000 codestream and sends them to the client. The client decodes the received packets and renders the image. Through a graphic user interface (GUI) on the client-side, a user can request different regions, resolutions, components, and number of layers at any time. To minimize the number of transmitted packets and maximize the responsiveness of interactive image browsing, the server assumes that the client employs a cache to hold data from previous requests, and maintains a cache model to keep track of the client cache. If a request is found to contain cached data, the server does not re-send that portion of the data.

The experimental results described below were obtained using the same codestreams employed in the experiments described in Section 5.1 above. As described there, these codestreams were created using 5 levels of 9/7 DWT, precincts of size 128 *×* 128 at each resolution level, and the CPRL progression order. All codestreams were JPEG 2000 Part 1 compliant. All JPIP experiments were conducted with an unmodified version of kdu_server (from Kakadu v6.4). The kdu_show client was adapted to specifically request only the number of quality layers commensurate with the codestream construction (single-layer, 6-layer, 24-layer) and the resolution level currently being displayed by the client. All client/server communications were JPIP compliant.

Table 10 reports the number of bytes transmitted via JPIP for 4 different images and 3 different visually lossless coding methods. The dimensions (size) of each image are included in the table as well. The rows in the table correspond to different images, while the 3 rightmost columns correspond to the different coding methods. The same sequence of browsing operations was issued by the JPIP client for each image and each coding method. In particular, for each codestream under test, the (*LL*, 5) image (*p* = 0.03125) was first requested. This resulted in an overview of the entire image being displayed in a window of size 0.03125 times the dimensions of the image under test (e.g., 794 *×* 822 for pathology 3.) This initial window size was maintained throughout the browsing session of the image under test. Following the initial request, 4 different locations in the image were requested with progressively higher resolution scales (*p* = 0.15, 0.36, 0.72, 1.0). Three pan operations were included after each new location request. As mentioned above, only the appropriate layers were transmitted for the 6-layer and 24-layer methods. As expected, in each case, the number of bytes required by the 6-layer method was significantly less than that required for the single-layer method. In turn, the 24-layer method resulted in significant savings over the 6-layer method. Averaged over the four images, the number of transmitted bytes for the single-layer method was 9180.9 KB, while the number of transmitted bytes for the 6-layer and 24-layer methods was 6279.2 KB and 4679.2 KB, representing a decrease of 31.61% and 49.03% over the single-layer method, respectively. It is clear from these results that the codestreams encoded by the 6-layer and 24-layer methods require considerably less bandwidth than a codestream optimized only for full resolution display.

**Figure 8:**
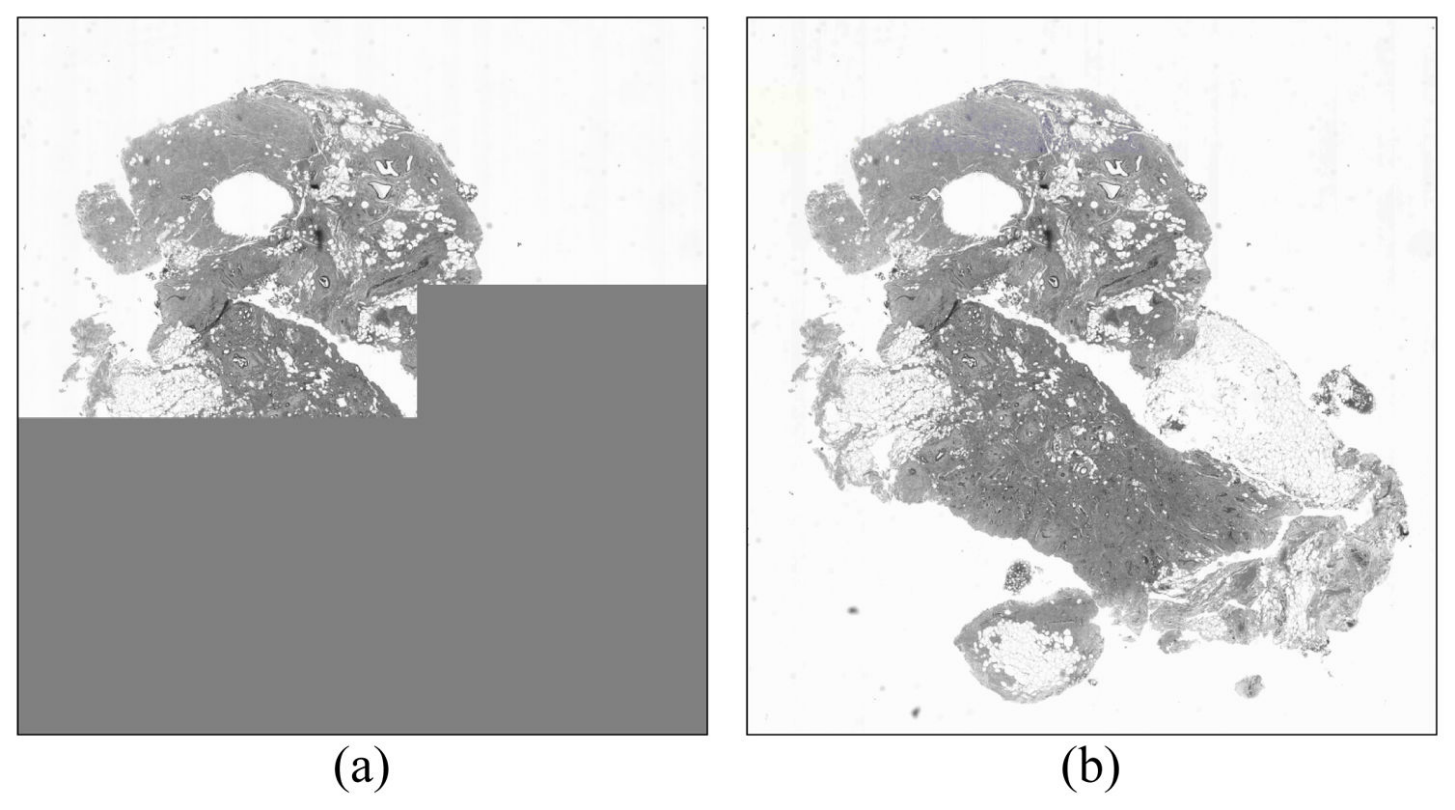
Images rendered while the image data are being retrieved via a JPIP client. (a) single-layer method at 131.2 KB and (b) 24-layer method at 130.4 KB.

As mentioned in Section III, layers are essential to effective browsing of remote images. Specifically, no quality progressivity is possible for single-layer codestreams. Figure 8 demonstrates this via example images obtained via JPIP browsing of a single-layer file vs. a 24-layer file for roughly the same number of transmitted bytes. While neither image is (yet) visually lossless^1^, for the number of bytes transmitted up to the moment of rendering in the figure, the advantage of progressive transmission is readily apparent. Using the image from the 24-layer file, the user may be able to make their next request, thus preempting the current request, without waiting for the rest of the data to be transmitted. This can further reduce bandwidth, but was not used to obtain the values in Table 10.

## 6. Conclusions

This paper presents a multi-resolution visually lossless image coding method using JPEG2000, which uses visibility threshold values measured for downsampled JPEG2000 quantization distortion. This method is implemented via JPEG2000 layers. Each layer is constructed such that the maximum quantization error in each codeblock is less than the appropriate visibility threshold at the relevant display resolution. The resulting JPEG2000 Part 1 compliant coding method allows for visually lossless decoding at resolutions natively supported by the wavelet transform as well as arbitrary intermediate resolutions, using only a fraction of the full-resolution codestream. In particular, when decoding the full field of view of a very large image at various reduced resolutions, the proposed 6-layer method reduces the amount of data that must be accessed and decompressed by 30 to 50 percent compared to that required by single-layer visually lossless compression. This is true despite the fact that the single-layer method does not access nor decode data from unnecessary high frequency subbands. The 24-layer method provides additional improvements over the 6-layer method ranging from 25 to 40 percent. This in turn, brings the gains of the 24-layer method over the single-layer method into the range of 55 to 65 percent. Stated another way, the gain in the amount of data access and decompressed (effective compression ratio) is improved by a factor of more than 2. These gains are born out in a remote image browsing experiment using digital pathology images and satellite images. In this experiment, JPIP is used to browse limited fields of view at different resolutions while employing zoom and pan. In this scenario, the proposed method exhibits gains similar to those described above, with no adverse effects on visual image quality.

## Figures and Tables

**Table 1:** Linear parameters *u***_b_** and *v***_b_** for luminance components.

Subband	*u*_b_	*v*_b_
(HH,1)	105.67 *×* 10^−4^	4.85
(HL/LH,1)	46.03 *×* 10^−4^	1.98
(HH,2)	19.94 *×* 10^−4^	0.92
(HL/LH,2)	13.84 *×* 10^−4^	0.64
(HH,3)	11.04 *×* 10^−4^	0.51
(HL/LH,3)	10.83 *×* 10^−4^	0.50
(HH,4)	10.16 *×* 10^−4^	0.47
(HL/LH,4)	7.75 *×* 10^−4^	0.36
(HH,5)	7.91 *×* 10^−4^	0.36
(HL/LH,5)	7.16 *×* 10^−4^	0.33

**Table 2:** Visibility thresholds for chrominance components.

Subband	Cb	Cr
(HH,1)	24.40	15.60
(HL/LH,1)	13.90	6.40
(HH,2)	14.91	7.35
(HL/LH,2)	6.39	2.55
(HH,3)	10.89	2.65
(HL/LH,3)	4.03	1.23
(HH,4)	4.47	1.27
(HL/LH,4)	2.97	0.72
(HH,5)	1.10	0.65
(HL/LH,5)	1.05	0.60
(LL, 5)	1.19	0.66

**Table 3:** Parameters for *t*_(_*_LL_*_,_*_k_*_)_ for luminance subband (*LL*, *k*).

k	u_(LL,k)_	v_(LL,k)_
0	0.2311	2.0170
1	0.3081	0.8095
2	0.0802	0.8270
3	0.1032	0.5893
4	0.0309	0.6848
5	0.0128	0.5923

**Table 4:** Thresholds *t*_(_*_LL_*_,_*_k_*_)_ for chrominance subband (*LL*, *k*).

*k*	Cb	Cr
0	4.73	4.50
1	3.78	3.40
2	2.45	2.12
3	2.31	1.85
4	1.60	1.00
5	1.19	0.66

**Table 5:** Visibility thresholds *t̄***_b_**_,_*_n_* for downscaling of the full resolution image (*LL*, 0) for typical subband variance values — Luminance (Y): 2000 for LL; 50 for HL/LH and HH. Chrominance (Cb and Cr): 150 for LL; 5 for HL/LH and HH.

Component	Subband	60% (*n* = 1)	72% (*n* = 2)	86.4% (*n* = 3)	100% (*n* = 4)
Y	(*LL*, 5)	0.89	0.83	0.78	0.63
	(*HL*/*LH*, 5)	0.70	0.59	0.57	0.37
	(*HL*/*LH*, 4)	0.72	0.65	0.59	0.40
	(*HL*/*LH*, 3)	1.00	0.87	0.79	0.55
	(*HL*/*LH*, 2)	1.88	1.65	1.35	0.71
	(*HL*/*LH*, 1)	5.39	4.90	3.15	2.21
	(*HH*, 5)	0.7	0.63	0.60	0.40
	(*HH*, 4)	0.75	0.68	0.65	0.52
	(*HH*, 3)	1.13	0.98	0.90	0.57
	(*HH*, 2)	4.20	2.95	2.05	1.02
	(*HH*, 1)	17.47	12.50	9.50	5.38

Cb	(*LL*, 5)	1.50	1.32	1.21	1.19
	(*HL*/*LH*, 5)	1.32	1.20	1.12	1.05
	(*HL*/*LH*, 4)	3.52	3.18	3.16	2.97
	(*HL*/*LH*, 3)	6.24	5.00	4.35	4.03
	(*HL*/*LH*, 2)	10.13	8.41	7.63	6.39
	(*HL*/*LH*, 1)	55.60	41.70	27.80	13.90
	(*HH*, 5)	2.20	1.54	1.45	1.10
	(*HH*, 4)	6.16	5.01	4.50	4.47
	(*HH*, 3)	14.70	14.30	11.59	10.89
	(*HH*, 2)	19.49	18.05	15.10	14.91
	(*HH*, 1)	97.60	73.20	48.80	24.40

Cr	(*LL*, 5)	1.05	0.98	0.95	0.66
	(*HL*/*LH*, 5)	1.08	1.01	1.05	0.60
	(*HL*/*LH*, 4)	1.27	1.23	1.10	0.72
	(*HL*/*LH*, 3)	1.98	1.58	1.45	1.23
	(*HL*/*LH*, 2)	4.78	3.50	2.71	2.55
	(*HL*/*LH*, 1)	25.60	19.20	12.80	6.40
	(*HH*, 5)	1.12	0.95	0.87	0.65
	(*HH*, 4)	2.45	1.80	1.36	1.27
	(*HH*, 3)	6.44	3.76	3.11	2.65
	(*HH*, 2)	14.47	12.51	11.36	7.35
	(*HH*, 1)	62.40	46.80	31.20	15.60

**Table 6:** Visibility thresholds *t̄***_b_**_,_*_n_* for the LL subband for typical subband variance values — Luminance (Y): 2000. Chrominance (Cb and Cr): 150.

Component	Subband	60% (*n* = 1)	72% (*n* = 2)	86.4% (*n* = 3)	100% (*n* = 4)
Y	(*LL*, 4)	0.92	0.89	0.85	0.79
	(*LL*, 3)	1.06	0.98	0.97	0.93
	(*LL*, 2)	1.35	1.22	1.17	1.09
	(*LL*, 1)	2.50	2.25	1.88	1.83
	(*LL*, 0)	7.33	4.65	3.85	2.78

Cb	(*LL*, 4)	2.15	1.89	1.72	1.60
	(*LL*, 3)	2.60	2.50	2.45	2.31
	(*LL*, 2)	3.60	3.13	2.75	2.45
	(*LL*, 1)	4.55	4.10	4.05	3.78
	(*LL*, 0)	8.80	7.50	6.30	4.73

Cr	(*LL*, 4)	1.51	1.40	1.38	1.00
	(*LL*, 3)	2.08	2.00	1.95	1.85
	(*LL*, 2)	3.27	2.70	2.45	2.12
	(*LL*, 1)	4.4	3.90	3.60	3.40
	(*LL*, 0)	5.25	5.10	4.90	4.50

**Table 7:** Average bits-per-pixel (bpp) decoded with respect to the full-resolution dimensions.

Resolution	Single-layer	6-layer	24-layer
(*LL*, 5), *n* = 1	0.00983	0.00597	0.00448
(*LL*, 5), *n* = 2	0.00983	0.00597	0.00529
(*LL*, 5), *n* = 3	0.00983	0.00597	0.00529
(*LL*, 5), *n* = 4	0.00983	0.00597	0.00598
(*LL*, 4), *n* = 1	0.02737	0.01370	0.00955
(*LL*, 4), *n* = 2	0.02737	0.01370	0.01078
(*LL*, 4), *n* = 3	0.02737	0.01370	0.01156
(*LL*, 4), *n* = 4	0.02737	0.01370	0.01376
(*LL*, 3), *n* = 1	0.08436	0.04377	0.02823
(*LL*, 3), *n* = 2	0.08436	0.04377	0.03205
(*LL*, 3), *n* = 3	0.08436	0.04377	0.03535
(*LL*, 3), *n* = 4	0.08436	0.04377	0.04416
(*LL*, 2), *n* = 1	0.26834	0.15365	0.09468
(*LL*, 2), *n* = 2	0.26834	0.15365	0.10177
(*LL*, 2), *n* = 3	0.26834	0.15365	0.13000
(*LL*, 2), *n* = 4	0.26834	0.15365	0.15566
(*LL*, 1), *n* = 1	0.84707	0.58476	0.35592
(*LL*, 1), *n* = 2	0.84707	0.58476	0.40256
(*LL*, 1), *n* = 3	0.84707	0.58476	0.43872
(*LL*, 1), *n* = 4	0.84707	0.58476	0.59581
(*LL*, 0), *n* = 1	1.68468	1.69689	1.07471
(*LL*, 0), *n* = 2	1.68468	1.69689	1.12744
(*LL*, 0), *n* = 3	1.68468	1.69689	1.41057
(*LL*, 0), *n* = 4	1.68468	1.69689	1.73476

**Table 8:** Bitrates (bpp) of the proposed 6-layer method vs. the single-layer method with added layers.

Image	Proposed 6-layer	Single-layer
*pathology 3*	1.1429	1.1441
*satellite 6*	2.1403	2.1425

**Table 9:** t-test results (test value= 1/3, *N* = 15).

Session (*p*)	Mean	Standard Deviation	Standard Error Mean	t-score	Significance (2-tailed)	95% confidence interval	
						Lower	Upper
0.03125	0.3307	0.05688	0.01469	−0.179	0.860	0.2992	0.3622
0.09	0.3347	0.04779	0.01234	0.111	0.913	0.3082	0.3611
0.25	0.3540	0.03996	0.01032	2.006	0.065	0.3319	0.3761
0.72	0.3453	0.03021	0.00780	1.543	0.145	0.3286	0.3621

**Table 10:** Transmitted bytes for the three visually lossless coding methods while remotely browsing compressed images.

Image	Dimensions	Single-layer	6-layer	24-layer
pathology 1	22040 *×* 21320	7966.6 KB	5691.4 KB	4222.3 KB
pathology 3	25408 *×* 26288	7917.0 KB	5133.1 KB	3855.9 KB
pathology 5	39912 *×* 29032	10699.8 KB	7199.5 KB	5190.9 KB
satellite 2	*XXX × YYY*	10140.3 KB	7092.6 KB	5447.5 KB

## Footnotes

1 The top portion of the single-layer image is visually lossless.
